# Comparative genomic analysis of the family *Iridoviridae*: re-annotating and defining the core set of iridovirus genes

**DOI:** 10.1186/1743-422X-4-11

**Published:** 2007-01-19

**Authors:** Heather E Eaton, Julie Metcalf, Emily Penny, Vasily Tcherepanov, Chris Upton, Craig R Brunetti

**Affiliations:** 1 Trent University, Department of Biology, 1600 East Bank Dr., Peterborough, ON, Canada; 2 Department of Biochemistry and Microbiology, University of Victoria, Victoria, BC, Canada

## Abstract

**Background:**

Members of the family *Iridoviridae *can cause severe diseases resulting in significant economic and environmental losses. Very little is known about how iridoviruses cause disease in their host. In the present study, we describe the re-analysis of the *Iridoviridae *family of complex DNA viruses using a variety of comparative genomic tools to yield a greater consensus among the annotated sequences of its members.

**Results:**

A series of genomic sequence comparisons were made among, and between the *Ranavirus *and *Megalocytivirus *genera in order to identify novel conserved ORFs. Of these two genera, the *Megalocytivirus *genomes required the greatest number of altered annotations. Prior to our re-analysis, the Megalocytivirus species orange-spotted grouper iridovirus and rock bream iridovirus shared 99% sequence identity, but only 82 out of 118 potential ORFs were annotated; in contrast, we predict that these species share an identical complement of genes. These annotation changes allowed the redefinition of the group of core genes shared by all iridoviruses. Seven new core genes were identified, bringing the total number to 26.

**Conclusion:**

Our re-analysis of genomes within the *Iridoviridae *family provides a unifying framework to understand the biology of these viruses. Further re-defining the core set of iridovirus genes will continue to lead us to a better understanding of the phylogenetic relationships between individual iridoviruses as well as giving us a much deeper understanding of iridovirus replication. In addition, this analysis will provide a better framework for characterizing and annotating currently unclassified iridoviruses.

## Background

Iridoviruses are large DNA viruses (~120–200 nm in diameter) that replicate in the cytoplasm of infected cells. Iridovirus genomes are circularly permuted and terminally redundant, and range in size from 105 to 212 kbp [1,2]. The family *Iridoviridae *is currently subdivided into five genera:*Chloriridovirus, Iridovirus, Lymphocystivirus, Megalocytivirus*, and *Ranavirus *[3].

Iridoviruses have been found to infect invertebrates and poikilothermic vertebrates, including amphibians, reptiles, and fish [4]. Iridovirus infections produce symptoms that range from subclinical to very severe, which may also result in significant mortality [5-9]. The high pathogenicity associated with some members of the iridovirus family has had a significant impact on modern aquaculture, fish farming, and wildlife conservation. For example, systemic iridovirus infections have been found in economically important freshwater and marine fish species worldwide. In addition, iridovirus infections have been implicated in amphibian population declines, representing a set of emerging infectious diseases whose spread has been accelerated by human activities [10-14].

Despite the economic and ecological significance of iridoviruses, very little is currently known about their molecular biology. One approach towards gaining a deeper understanding of iridoviral pathogenesis is to investigate the core set of essential genes conserved among all members of the family. The genomes of twelve iridoviruses, including at least one from each genus, have been completely sequenced (Table 1). According to the previously published annotations, these genomes contained only 19 core genes associated with a variety of viral activities: transcriptional regulation, DNA metabolism, protein modification, and viral structure. Definition of this core set of genes also highlights those genes that are conserved across some, but not all, genera, and unique genes found within a single species. These non-core genes may be involved in specific virus-host interactions, enhancement of virus replication, and augmented pathogenesis in certain species.

**Table 1:** Iridovirus Genomes

Virus	Abbreviation	Genus	Genome Size (bp)	# ORFs*	GenBank accession #	Ref
Frog virus 3	FV3	Ranavirus	105903	97	AY548484	[27]
Tiger frog virus	TFV	Ranavirus	105057	103	AF389451	[1]
Ambystoma tigrinum virus	ATV	Ranavirus	106332	92	AY150217	[30]
Grouper iridovirus	GIV	Ranavirus	139793	139	AY666015	[21]
Singapore grouper iridovirus	SGIV	Ranavirus	140131	139	AY521625	[22]
Lymphocystis disease virus 1	LCDV-1	Lymphocystivirus	102653	108	L63545	[34]
Lymphocystis disease virus China	LCDV-China	Lymphocystivirus	186250	178	AY380826	[24]
Infectious spleen and kidney necrosis virus	ISKNV	Megalocytivirus	111362	117	AF371960	[20]
Rock bream iridovirus	RBIV	Megalocytivirus	112080	116	AY532606	[19]
Orange-spotted grouper iridovirus	OSGIV	Megalocytivirus	112636	116	AY894343	[18]
Invertebrate iridescent virus 6	IIV-6	Iridovirus	212482	211	AF303741	[2]
Invertebrate iridescent virus 3	IIV-3	Chloriridovirus	191100	126	DQ643392	[26]

*The number of ORFs in each viral genome reflects the results of the analysis done in this paper

Despite the growing number of sequenced iridovirus genomes, no systematic comparative genomic analysis of the family has yet been performed. Thus, annotation of these genomes has been performed without standardization and has so far been guided primarily by the position of start/stop codons rather than the presence of homologous sequences. As a result, some long overlapping potential ORFs have been automatically designated as coding sequences, and smaller homologous ORFs overlooked. In this paper, we have taken a comparative genomics approach to re-examine the annotation of all twelve iridovirus genomes, using the Viral Orthologous Clusters (VOCs) [15] and Viral Genome Organizer (VGO) [16] software. These re-annotated genomes were then analysed further, both to define the core set of iridovirus genes more accurately, and to provide a deeper understanding into the phylogenetic relationship between individual iridovirus species.

## Results & discussion

### Re-annotation of Iridovirus genomes

One objective of this project was to demonstrate the application of comparative genomics to annotating viral genomes, particularly those that have been poorly characterized experimentally. In an earlier study, we utilized comparative genomics to identify previously unannotated small viral ORFs in the *Poxviridae *[17]. Here, we focused our analysis on the *Iridoviridae *family, which represents a challenge in genome annotation since there is little experimental evidence available to confirm gene expression. Another problem is that iridovirus promoter elements have not been well characterized, and thus cannot be used as a reliable criterion for assigning ORFs. These combined factors made previous iridovirus gene annotation a somewhat arbitrary process, resulting in closely related iridovirus species with dramatic differences in their genomic annotations. Therefore, we decided to analyse all members of this family using a standardized comparative genomics approach, using the fact that ORFs that are conserved in more than one divergent species are likely to be functional genes.

Analysis was begun with the *Megalocytivirus *genus, which contains three sequenced genomes: infectious spleen and kidney necrosis virus (ISKNV), rock bream iridovirus (RBIV), and orange-spotted grouper iridovirus (OSGIV). These three viruses display a co-linear arrangement of genes with an overall DNA sequence identity of greater than 90%. In the analysis of this genus, differences in gene content were examined in detail. Dotplots were used to determine presence of orthologous DNA and a variety of BLAST searches and the VGO genome visualization software were used to determine the reason (frameshifts, extra stop codons) behind the apparent absence of some ORFs.

Using this approach, a substantial number of ORFs were either added to, or deleted from members of the *Megalocytivirus *genus (Table 2). OSGIV and RBIV share 99% DNA sequence identity, and thus are probably different strains of the same virus; however, previous annotation described only 82 out of 118 total annotated ORFs shared by the two genomes [18,19]. After our re-analysis, the RBIV and OSGIV genomes had an identical complement of annotated genes. Furthermore, this re-annotated ISKNV genome contained 110 ORFs orthologous with both RBIV and OSGIV (compared to 71 in the old annotation.) (Table 2) [18,20].

**Table 2:** Re-annotation of the *Megalocytivirus *genus

**ISKNV**^a^	**Start**	**Stop**^c^	**aa**^d^	**RBIV**^a^	**Start**	**Stop**^c^	**aa**^d^	**OSGIV**^a^	**Start**	**Stop**^c^	**aa**^d^
1L	1270	134	378	1L	1270	134	378	1L	1270	134	378
2R	1394	2044	216	**2R**^b^	**1394**	**1597/1781**	**67**	2R	1394	1789	131
-	-	-	-	3R	1841	2056	71	3R	1849	2064	71
3L	2634	2077	185	4L	2605	2102	167	4L	2613	2110	167
4L	2890	2681	69	5L	2800	2624	58	5L	2808	2632	58
5L	3648	2893	251	6L	3541	2876	221	6L	3548	2883	221
6L	5155	3794	453	7L	5147	3786	453	7L	5154	3793	453
7L	6631	5174	485	8L	6621	5164	485	8L	6628	5171	485
8R	6669	8246	525	9R	6692	8239	515	9R	6699	8246	515
9R	8342	8503	53	10R	8335	8496	53	10R	8342	8503	53
10L	9054	8662	130	11L	9047	8655	130	**11L/12L**^b^	**9055**	**8849/8662**	**130**
11L	9311	9051	86	**11.5L**	**9304**	**9044**	**86**	13L	9312	9052	86
12R	9330	9659	110	12R	9323	9655	110	14R	9331	9663	110
13R	9669	11054	461	13R	9662	11059	465	15R	9670	11067	465
14R	11309	12268	319	14R	11314	12288	324	16R	11322	12296	324
15R	12278	13069	263	15R	12298	13089	263	17R	12302	13093	263
16L	13716	13129	195	16L	13733	13146	195	18L	13738	13151	195
17L	14095	13718	125	17L	14086	13748	112	19L	14088	13753	111
**17.5R**	**14089**	**14325**	**78**	18R	14171	14410	79	20R	14094	14351	85
**18.5L**^b^	**14563**	**14233**	**109**	19L	14648	14472	58	21L	14607	14431	58
19R	14579	17425	948	20R	14664	17510	948	22R	14623	17469	948
20L	17642	17454	62	21L	17756	17574	60	23L	17715	17533	60
21L	17900	17778	40	**21.5L**	**18014**	**17892**	**40**	24L	17973	17851	40
22L	19489	17990	499	22L	19714	18104	536	25L	19715	18063	550
23R	19562	22132	856	**23R**	**19787**	**22204**	**805**	26R	19788	22922	1044
24R	22300	23238	312	26R	23035	23973	312	27R	23207	24145	312
25R	23354	23779	141	27R	23997	24380	127	28R	24169	24696	175
26L	24145	23822	107	27.5L	24697	24377	106	29L	25013	24693	106
27L	25063	24167	298	28L	25615	24719	298	30L	25931	25035	298
28L	28559	25080	1159	29L	29138	25632	1168	31L	29454	25948	1168
29L	28814	28593	73	**29.5L**^b^	**29362**	**29087/29145**	**91**	32L	29682	29461	73
**31.5L**	**29414**	**28884**	**176**	**30.5L**	**29957**	**29430**	**175**	**33.5L**	**30277**	**29750**	**175**
32R	29447	30061	204	31R	29990	30622	210	34R	30310	30942	210
33L	31079	30138	313	32L	31654	30713	313	35L	31935	31033	300
34R	31144	34278	1044	33R	31700	34861	1053	36R	32018	35176	1052
35L	35508	34360	382	34L	36067	34934	377	37L	36382	35249	377
36R	35546	36601	351	**35.5R**	**36061**	**37113**	**350**	38R	36376	37431	351
37L	37950	36598	450	37L	38219	37110	369	39L	38777	37428	449
38L	39395	37959	478	38L	39974	38469	501	40L	40225	38786	479
39R	39439	40311	290	**39.5R**^b^	**40012**	**40506/40857**	**164**	41R	40290	41168	292
40L	41443	40304	379	41L	41995	40850	381	42L	42306	41161	381
41L	42788	41445	447	42L	43346	41997	449	43L	43657	42308	449
42R	42803	43396	197	**43R**	**43361**	**43959**	**198**	44R	43672	44271	199
43L	43842	43480	120	**43.5L**	**44405**	**43975**	**142**	45L	44717	44355	120
44L	44645	43845	266	44L	45208	44408	266	46L	45524	44724	266
45L	45564	44650	304	45L	46127	45213	304	47L	46443	45529	304
46L	46241	45558	227	46L	46804	46121	227	48L	47120	46437	227
47R	46401	46664	87	47R	47150	46887	87	49R	47280	47543	87
48R	46661	47005	114	**47.5R**^b^	**47224**	**47433/47588**	**69**	50R	47540	47893	117
49R	47021	47191	56	**48.5R**	**47604**	**47774**	**56**	51R	47909	48079	56
50L	47678	47250	142	49L	48270	47842	142	52L	48575	48147	142
51R	47733	47864	43	-	-	-	-	-	-	-	-
52L	48403	47951	150	50L	48999	48547	150	53L	49306	48854	150
53R	48405	48620	71	51R	49001	49195	64	54R	49308	49502	64
54L	49559	48633	308	52L	50173	49229	314	55L	50480	49536	314
55L	50508	49582	308	53L	51137	50196	313	56L	51444	50503	313
56L	51166	50519	215	54L	51795	51148	215	57L	52102	51455	215
57L	51433	51173	86	55L	52062	51802	86	58L	52369	52109	86
**59L**	**52414**	**51749**	**221**	56L	52839	52327	170	59L	53146	52634	170
61L	53162	52359	267	57L	53709	52903	268	60L	54016	53210	268
62L	56785	53159	1208	58L	57467	53706	1253	**61L/62L**^b^	**55131**	**54013/53893**	**372**
63L	59875	57227	882	59L	60567	57919	882	63L	60876	58228	882
64L	61393	59918	491	**60L**^b^	**62102**	**60855/60635**	**415**	64L	62416	60944	490
65L	61900	61439	153	61L	62611	62144	155	65L	62928	62458	156
66L	63025	61982	347	62L	63744	62662	360	66L	64061	62979	360
67L	63855	63271	194	**63.5L**	**64446**	**63865**	**193**	**67.5L**	**64763**	**64182**	**193**
68L	65329	63896	477	64L	65917	64484	477	69L	66234	64801	477
69L	66001	65336	221	**65L**^b^	**66661**	**66215/65929**	**148**	70L	66977	66246	243
70L	66331	66101	76	-	-	-	-	-	-	-	-
71L	68042	66432	536	68L	68529	67120	469	71L	68973	67417	518
72R	68173	69177	334	-	-	-	-	-	-	-	-
73R	69203	69622	139	**69R**^b^	**68717**	**69191/69135**	**157**	72R	69078	69497	139
74R	69669	70682	337	70R	69184	70203	339	73R	69546	70568	340
75L	71043	70777	88	71L	70573	70304	89	74L	70938	70669	89
76L	74017	71045	990	72L	73541	70575	988	75L	73912	70940	990
77R	74035	75369	444	**73R**	**73559**	**74893**	**444**	76R	73930	75264	444
78R	75366	75830	154	75R	74890	75354	154	77R	75261	75725	154
79L	76053	75832	73	76L	75580	75356	74	78L	75951	75727	74
-	-	-	-	77R	75664	76137	157	79R	76039	76512	157
80L	76368	76165	67	-	-	-	-	-	-	-	-
81R	76367	76864	165	78R	76150	76647	165	80R	76525	77022	165
82L	78007	76901	368	79L	77802	76696	368	81L	78177	77071	368
83R	78152	78418	88	80R	77827	78225	132	82R	78202	78600	132
84L	79881	78526	451	81L	79556	78252	434	83L	79931	78627	434
85R	79884	80486	200	82R	79643	80173	176	84R	80018	80548	176
86R	80483	80947	154	**82.5R**	**80170**	**80637**	**155**	**84.5L**	**80545**	**81012**	**155**
87R	80940	81710	256	83R	80603	81400	265	85R	80978	81775	265
88R	81717	83720	667	84R	81503	83425	640	**86R/87R**^b^	**82234**	**83805/82279**	**523 **
**90.5L**	**84663**	**83701**	**320**	85L	84457	83630	275	88.5L	84811	83786	341
93L	85786	84860	308	86L	85504	84578	308	90L	85918	84992	308
94L	86296	85796	166	87L	86014	85514	166	91L	86428	85928	166
95L	87481	86321	386	88L	87202	86039	387	92L	87616	86453	387
96L	88298	87489	269	**89.5L**^b^	**87601**	**87218/87210**	**127**	93L	88361	87624	245
**97.5L**	**88723**	**88232**	**163**	90L	88443	87952	163	94L	88857	88366	163
99L	89097	88774	107	91L	88814	88491	107	95L	89229	88906	107
100L	89689	89144	181	92L	89515	88868	215	96L	90024	89377	215
101L	90251	89736	171	**93.5L**	**89999**	**89470**	**175**	97L	90508	89993	171
102R	90311	91753	480	94R	90068	91513	481	98R	90577	92022	481
103R	91760	92161	133	**95.5R**	**91477**	**91935**	**152**	99R	92029	92442	137
104R	92215	92991	258	96R	91994	92773	259	100R	92501	93280	259
105R	92993	93358	121	97R	92775	93146	123	101R	93282	93653	123
-	-	-	-	98R	93240	94127	295	102R	93747	94634	295
106L	94501	93482	339	99L	95042	94221	273	103L	95548	94676	290
**108.5L**	**95093**	**94494**	**199**	**100.5L**	**95699**	**95068**	**209**	**104.5L**	**96217**	**95574**	**213**
109L	97950	95185	921	101L	98557	95792	921	106L	99060	96298	920
110R	97997	98152	51	**101.5L**	**98609**	**98764**	**51**	107R	99113	99268	51
111L	99039	98149	296	102L	99657	98761	298	**108L**^b^	**99885**	**99265/99849**	**206**
112R	99059	99802	247	**103R**^b^	**99677**	**100426/100422**	**249**	109R	100183	100926	247
113R	99937	100290	117	**104.5R**	**100493**	**100909**	**138**	110R	100998	101414	138
114L	103159	100334	941	**106L**^b^	**103615**	**102539/100953**	**358**	111L	104041	101594	815
115R	103203	104213	336	**108.5R**	**104050**	**104781**	**243**	112R	104476	105486	336
116R	104219	105667	482	110R	105060	106493	477	113R	105547	106701	384
117L	106395	105721	224	111L	107221	106547	224	114L	107652	106978	224
118L	108093	106723	456	112L	108913	107549	454	115L	109299	107986	437
119R	108105	108392	95	113R	108931	109104	57	116R	109369	109656	95
120R	108424	108933	169	114R	109248	109637	129	117R	109687	110193	168
121L	109584	108934	216	**115L**	**110419**	**109756**	**220**	118L	110849	110214	211
122R	109594	110313	239	116R	110429	111148	239	119R	110859	111578	239
123R	110391	110576	61	117R	111226	111420	64	120R	111656	111850	64
124L	111351	110665	228	118L	112037	111576	153	121L	112625	111939	228

^a^ORFs that have been added or altered are highlighted in bold. If a previously annotated ORF is not listed in the table, it has been deleted.

^b^Potentially frameshifted ORF

^c^Where an ORF has a potential sequencing error resulting in a frameshift mutation, 2 stop codons are provided in the format X/Y. The first number represents the actual physical stop in the reported sequence. The second number is the proposed stop if a sequencing error occurred.

^d^Length of ORF in amino acids

In the process of re-examining these genomes, we annotated a number of genes containing apparent frameshift mutations between species. In RBIV we annotated ten genes with potential frameshift mutations, while OSGIV had four such genes (Table 2). All of the genes containing potential frameshift mutations had orthologs in the other two members of the *Megalocytivirus *genus (Table 2). In some cases, these mutations may be the result of natural mutations within the viruses; however, it is also possible that these apparent frameshift mutations are actually sequencing errors. For both RBIV and OSGIV, PCR primers based on the ISKNV sequence were used to amplify genomic fragments, which were subsequently sequenced [18,19]. It is possible that errors were introduced during the PCR process, leading to apparent frameshifts in the reported sequence. It is interesting to note that the genomic sequence of ISKNV (sequenced using subcloned fragments rather than PCR products) [20], had significantly fewer annotation changes made during our re-analysis. Though we have not experimentally proven that the frameshift mutations in OSGIV and RBIV are the result of sequencing errors, it would be useful to focus future sequencing efforts on these regions, to determine if the reported sequences are indeed correct.

After re-annotating the *Megalocytivirus *genus, we applied the same comparative genomic analysis to the *Ranavirus *genus. The genus contains five sequenced members divided into two groups, each with a high degree of sequence conservation and a co-linear arrangement of genes. The first group is comprised of frog virus 3 (FV3), tiger frog virus (TFV), and *Ambystoma tigrinum *virus (ATV). The second group contains Singapore grouper iridovirus (SGIV) and grouper iridovirus (GIV).

The first step in the re-annotation of the *Ranavirus *genus was a comparative genomic analysis of FV3, TFV, and ATV. This resulted in an increase in the number of conserved annotated genes from 76 to 87 (Table 3). Subsequent re-analysis of the second *Ranavirus *group, containing SGIV and GIV, resulted in an increase from 131 to 138 conserved annotated ORFs (Table 4). It should be noted that two of the newly annotated ORFs, SGIV 0.5L and GIV 120.5L, appear to "wrap around", beginning at one end of the genome with the remainder of the ORF located at the opposite end [21,22]. These apparent "split ORFs" are actually the result of the circularly permutated iridovirus genome being represented as a linear genomic sequence, when the arbitrarily chosen start point happens to fall in the middle of an ORF [23].

**Table 3:** Re-annotation of FV3, TFV, and ATV of the *Ranavirus *genus

**FV3**^a^	**Start**	**Stop**^c^	**aa**^d^	**TFV**^a^	**Start**	**Stop**^c^	**aa**^d^	**ATV**^a^	**Start**	**Stop**^c^	**aa**^d^
1R	272	1042	256	105R	103809	104576	256	91R	104836	105606	256
2L	2611	1649	320	**2L**^b^	**1028**	**315/11**	**237**	1L	981	70	303
**2.5L**	**3488**	**2649**	**279**	**2.5L**	**1943**	**1065**	**292**	2L	1858	1019	279
3R	3418	4734	438	4R	1937	3151	404	3R	1892	3106	404
4R	4775	4957	60	5R	3190	3372	60	4R	3149	3331	60
5R	5390	6004	204	6R	3816	4418	200	-	-	-	-
6R	6007	6234	75	-	-	-	-	-	-	-	-
-	-	-	-	**6.5R**	**4411**	**4578**	**55**	-	-	-	-
**7.5L**	**7025**	**7411**	**128**	**7L**	**5452**	**5024**	**142**	5L	4416	3994	140
8R	7503	11384	1293	8R	5531	9415	1294	6R	4495	8379	1294
9L	14599	11753	948	9L	12599	9753	948	7L	11725	8879	948
10R	14615	15028	137	10R	12615	13028	137	8R	11741	12154	137
11R	15378	15590	70	11R	13380	13592	79	88L	102924	102712	70
12L	16549	15656	297	12L	14551	13658	297	87R	101753	102646	297
-	-	-	-	13L	14947	14747	66	86R	101169	101363	64
13R	17090	17296	68	**14.5R**^b^	**15041**	**15184/15247**	**47**	**85.5L**	**101128**	**100871**	**85**
14R	17311	17670	119	15R	15261	15620	119	84L	100856	100482	124
15R	17766	18734	322	16R	15716	16663	315	83R	100400	99474	308
16R	19014	19841	275	17R	16838	17665	275	**82.5L**	**98809**	**98438**	**123**
17L	21590	20082	502	18L	19414	17906	502	81R	96410	97918	502
18L	21864	21628	78	**18.5L**	**19687**	**19451**	**78**	**80.5R**	**96137**	**96373**	**78**
19R	21916	24471	851	**19R**	**19686**	**22271**	**861**	80L	96083	94086	665
20R	24519	24965	148	20R	22319	22774	151	79L	94038	93589	149
21L	25861	25202	219	21L	23657	23998	219	78R	92383	93042	219
22R	25991	28912	973	22R	23789	26716	975	77L	92253	89326	975
23R	29290	30438	382	23R	27093	28241	382	53R	58082	59230	382
24R	30821	31918	365	24R	28636	29733	365	54R	59613	60710	365
25R	32112	32900	262	25R	29930	30709	259	55R	62328	63335	335
-	-	-	-	26R	30778	30936	52	56R	63402	63500	32
**26R**^b^	**32967**	**33197**	**76**	27R	31033	31812	259	57R	63659	64438	259
-	-	-	-	28L	32190	32002	62	-	-	-	-
27R	33728	36640	970	29R	32345	35257	970	58R	64968	67880	970
28R	36689	37177	162	30R	35306	35794	162	59R	67929	68417	162
29L	37652	37356	98	31L	36122	35823	99	-	-	-	-
30R	37854	38006	50	-	-	-	-	-	-	-	-
31R	38068	38487	139	32R	36565	36984	139	60R	68786	69205	139
32R	38537	40426	629	33R	37098	39047	649	61R	69255	71471	738
33R	40509	40700	63	34R	39133	39324	63	62R	71555	71746	63
34R	40844	41164	106	**35.5R**	**39467**	**39787**	**106**	62.5R	71894	72235	113
35L	41717	41256	153	37L	40308	39772	178	63L	72576	72220	118
36L	42353	41256	365	**38.5L**^b^	**40938**	**40543/40367**	**131**	-	-	-	-
-	-	-	-	39R	41112	41246	44	-	-	-	-
37R	42749	43378	209	40R	41296	41952	218	64R	74110	74739	209
38R	43519	45216	565	41R	42091	43788	565	65R	74878	76575	565
39R	45322	45672	116	42R	43899	44249	116	66R	76682	76948	88
40R	45761	46309	182	43R	44335	44883	182	67R	77048	77671	207
-	-	-	-	44R	44973	45239	88	68R	77899	78039	46
41R	46691	50188	1165	45R	45270	48767	1165	69R	78111	81608	1165
**43.5L**^b^	**50940**	**51455/5684**	**171 **	46L	50362	49133	409	70L	82913	82152	253
45L	52348	51938	136	47L	50899	50489	136	71L	83450	83040	136
46L	52968	52723	81	48L	51411	50953	152	72L	84331	83504	275
47L	53509	53093	138	49L	51953	51537	138	73L	84874	84458	138
48L	53763	53512	83	50L	52207	51956	83	74L	85187	84804	127
49L	54621	53872	249	51L	52899	52315	194	75L	86776	85235	513
50L	55459	54770	229	52L	53876	53136	246	-	-	-	-
51R	55539	57224	561	53R	53956	55641	561	76R	86858	88543	561
52L	58548	57481	355	54L	56965	55898	355	52R	57441	57602	53
53R	58886	60454	522	55R	57301	58869	522	51L	57102	55522	526
54L	60899	60669	76	-	-	-	-	-	-	-	-
55L	62232	60937	431	56L	60615	59320	431	50R	53770	55065	431
-	-	-	-	57L	60772	60623	49	49R	53613	53762	49
56R	62320	62757	145	58R	60809	61213	134	48L	53576	53172	134
57R	62871	64367	498	59R	61254	62750	498	47L	53130	51634	498
-	-	-	-	60L	62888	62757	43	-	-	-	-
**58.5R**	**64819**	**65373**	**184**	61R	63264	63818	184	46L	50770	50216	184
59L	67014	65956	352	62L	65445	64387	352	45R	48676	49734	352
60R	67176	70217	1013	63R	65605	68646	1013	44L	48512	45471	1013
61L	70408	70226	60	-	-	-	-	-	-	-	-
-	-	-	-	64R	69029	69151	40	-	-	-	-
62L	74516	70851	1221	65L	72940	69281	1219	43R	41447	45112	1221
**62.5R**	**74515**	**74778**	**87**	**66.5R**	**72927**	**73202**	**91**	**42.5L**	**41460**	**41185**	**91**
63R	74895	75389	164	68R	73319	73813	164	42L	41068	40631	145
64R	75529	75816	95	**69.5R**	**73946**	**74209**	**87**	40L	40492	40205	95
65L	76373	76209	54	-	-	-	-	-	-	-	-
66L	76921	76370	183	**70.5L**	**75301**	**74685**	**204**	**38.5R**	**39094**	**36681**	**195**
67L	78139	76976	387	71L	76525	75362	387	38R	37876	39039	387
68R	78422	78709	95	72R	76785	76982	65	37bL	37592	37416	58
-	-	-	-	73L	77175	77020	51	36R	36736	36891	51
69R	78845	79111	88	74R	77244	77507	88	35L	36677	36411	88
70R	79129	79503	124	75R	77507	77902	131	**34.5L**	**36392**	**36018**	**124**
71R	79543	79776	77	76R	77942	78175	77	34L	35978	35742	78
72L	80549	79833	238	77L	78948	78232	238	32R	34970	35293	107
73L	81971	80997	324	78L	80299	79325	324	31R	33319	34311	330
74L	83258	82146	370	79L	81498	80506	330	30R	31947	33128	393
75L	83544	83290	84	80L	81809	81555	84	29R	31637	31891	84
76R	83607	83828	73	81R	81872	82093	73	28L	31574	31353	73
77L	84172	83825	115	82L	82437	82090	115	27R	31009	31356	115
78L	85395	84757	212	83L	83568	82894	224	-	-	-	-
79R	85531	87249	572	84R	83668	85386	572	26L	30729	28999	576
80L	88987	87872	371	85L	86988	85873	371	25R	27224	28345	373
81R	89043	89321	92	86R	87046	87324	92	24L	27168	26890	92
82R	89450	89923	157	87R	87454	87927	157	23L	26762	26289	157
-	-	-	-	88R	88138	88512	124	22L	25564	25277	95
83R	90373	91017	214	89R	88857	89501	214	21L	24912	24268	214
84R	91389	92126	245	90R	89903	90640	245	20L	23923	23141	260
85R	92201	92788	195	**91.5R**	**90715**	**91302**	**195**	19L	23066	22479	195
86L	93363	93178	61	92L	91943	91650	97	18R	21742	22119	125
87L	95533	93716	605	93L	94096	92279	605	17R	19571	21397	608
88R	95566	96018	150	94R	94129	94581	150	16L	19538	19086	150
89R	96086	97252	388	95R	94649	95845	398	15L	19018	17744	424
90R	97345	98736	463	96R	95938	97329	463	14L	17651	16260	463
91R	98860	100047	395	97R	97453	98640	395	13L	16136	14949	395
92R	100398	100637	79	98R	98927	99232	101	-	-	-	-
93L	100986	100819	55	99L	99593	99426	55	12R	14091	14246	51
94L	101563	101096	155	100R	100169	99702	155	11L	13512	13979	155
95R	101656	102747	363	101R	100180	101352	390	10L	13419	12325	364
96R	103549	104220	223	103R	102169	102840	223	89R	103279	103965	228
97R	104303	104716	137	104R	102923	103372	149	90R	104031	104444	137

^a^ORFs that have been added or altered are highlighted in bold. If a previously annotated ORF is not listed in the table, it has been deleted.

^b^Potentially frameshifted ORF

^c^Where an ORF has a potential sequencing error resulting in a frameshift mutation, 2 stop codons are provided in the format X/Y. The first number represents the actual physical stop in the reported sequence. The second number is the proposed stop if a sequencing error occurred.

^d^Length of ORF in amino acids

**Table 4:** Re-annotation of SGIV and GIV of the *Ranavirus *genus

**SGIV**^a^	**Start**	**Stop**^c^	**aa**^d^	**GIV**^a^	**Start**	**Stop**^c^	**aa**^d^
14L	12773	12348	141	**1.3L**	**2020**	**1595**	**141**
15L	13000	12821	59	**1.5L**	**2247**	**2068**	**59**
16L	14289	13048	413	2L	3536	2295	413
18R	14317	15174	285	3R	3564	4421	285
19R	15196	16224	342	4R	4443	5399	318
20L	17246	16278	322	5L	6421	5453	322
21L	17725	17306	139	6L	6900	6475	141
22L	18277	17777	166	7L	7486	6950	178
24L	18774	18319	151	8L	7983	7528	151
25L	20488	18956	510	9L	9682	8165	505
26R	20567	22267	556	10R	9761	11461	566
28L	23363	22350	337	11L	12559	11546	337
29L	24445	23447	332	12L	13659	12643	338
30L	25635	24610	341	13L	14850	13816	344
31L	27160	26144	338	14L	16384	15362	340
**32.5L**	**28666**	**27609**	**352**	15L	17904	16846	352
33L	29760	28726	344	16L	19010	17964	348
34L	30161	29823	112	**16.5L**	**19411**	**19073**	**112**
35L	31388	30261	375	17L	20638	19511	375
36L	32515	31526	329	18L	21835	20771	354
37L	33696	32668	342	19L	23016	21988	342
38L	34236	33724	170	20L	23556	23044	170
39L	37417	34262	1051	21L	26738	23583	1051
41L	37978	37547	143	22L	27296	26865	143
42R	38058	38285	75	-	-	-	-
43R	38285	40288	667	23R	27608	29605	665
45L	41090	40362	242	24L	30407	29679	242
46L	41866	41120	248	25L	31204	30467	245
47L	43063	41909	384	26L	32401	31247	384
48L	43489	43214	91	27L	32824	32549	91
49L	44002	43535	155	28L	33336	32857	159
50L	44695	44033	220	29L	34029	33367	220
51L	45563	44868	231	30L	34896	34201	231
52L	37997	35097	968	31L	37997	35097	966
54R	48777	49424	215	32R	38100	38747	215
55R	49447	50169	240	33R	38770	39492	240
56R	50198	50938	246	34R	39521	40261	246
57L	54510	51004	1168	35L	43833	40327	1168
59L	55000	54560	146	**35.5L**	**44323**	**43391**	**146**
60R	54967	57879	970	36R	44348	47200	950
61R	57914	58528	204	37R	47235	47849	204
62R	58593	59363	256	38R	47914	48708	264
64R	59415	61133	572	39R	48760	50478	572
65R	61268	61510	80	**39.5R**	**50614**	**50856**	**80**
66R	61603	61845	80	**39.7R**	**50949**	**51191**	**80**
67L	62482	61907	191	40L	51829	51254	191
68L	63334	62516	272	41L	52681	51863	272
69L	64967	63321	548	42L	54314	52668	548
70R	64994	65452	152	43R	54341	54799	152
71R	65483	66307	274	44R	54830	55654	274
72R	66404	67795	463	45R	55751	57142	463
73L	71185	67874	1103	46L	60532	57221	1103
74R	68472	68738	88	47R	57819	58085	88
75R	71239	71775	178	48R	60586	61122	178
76L	72715	71858	285	49L	62064	61207	285
77L	73747	72839	302	50L	63096	62188	302
-	-	-	-	51L	62944	62282	220
**78L+81L**^a^	**76809**	**76246/73855**	**984**	52L	66156	63202	984
82L	77592	76924	222	53L	66939	66271	222
83R	77672	79009	445	54R	67019	68356	445
84L	80193	79066	375	55L	69540	68413	375
85R	80251	80529	92	56R	69598	69876	92
86R	80591	81055	154	57R	69938	70402	154
87R	81385	82032	215	58R	70728	71375	215
88L	84187	82667	506	59L	73355	71835	506
89L	85420	84248	390	60L	74588	73416	390
90L	86627	85506	373	61L	75794	74673	373
91L	87886	86750	378	62L	77051	75915	378
92L	89216	88086	376	63L	78373	77240	377
93L	90497	89280	405	64L	79654	78437	405
95R	90635	91111	158	**64.5L**	**80265**	**79792**	**157**
96R	91148	91618	156	65R	80301	80771	156
97L	92774	91626	382	66L	81926	80778	382
98R	92428	93231	267	67R	81580	82383	267
99R	93244	93492	82	**67.5R**	**82380**	**82646**	**88**
101R	93753	94694	313	68R	82906	83847	313
102L	95007	94774	77	69L	84161	83928	77
103R	95092	95385	97	70R	84246	84539	97
104L	99252	95446	1268	71L	88406	84600	1268
105R	95498	95731	77	72R	84652	84885	77
107R	99308	100453	381	73R	88462	89088	208
111R	100766	101533	255	74R	89401	90168	255
112R	101588	102655	355	75R	90223	91326	367
114L	103050	102712	112	**77.5L**	**91721**	**91383**	**112**
115R	103122	103580	152	78R	91793	92251	152
116R	103700	104476	258	79R	92371	93147	258
117L	104733	104575	52	**79.5L**	**93401**	**93241**	**52**
118R	104795	105754	319	80R	93463	94422	319
119R	105799	106050	83	**80.5R**	**94467**	**94718**	**83**
120L	106525	106103	140	81L	95162	94779	127
121R	106615	106869	84	**81.5R**	**95291**	**95547**	**84**
122L	107599	106967	210	82L	96275	95643	210
123L	108740	107652	362	83L	97416	96328	362
124R	108863	109399	178	**83.5L**	**98976**	**97684**	**130**
125R	109474	110028	184	84R	98151	98705	184
126R	110101	110658	185	85R	98692	99330	212
127R	110731	111252	173	86R	99403	99924	173
128R	112041	115070	1009	87R	100788	103817	1009
129L	115490	115308	60	**87.5L**	**104245**	**103884**	**119**
131R	115749	116303	184	88R	104499	105053	184
132R	116321	117148	275	89R	105071	105898	275
134L	118498	117527	323	90L	107244	106273	323
135L	118885	118547	112	**90.5L**	**107631**	**107258**	**123**
136R	118946	119260	104	91R	107692	108006	104
137R	119282	120667	461	92R	108028	109413	461
138L	120907	120713	64	**92.3L**	**109653**	**109457**	**64**
139R	121013	121324	103	**92.5R**	**109757**	**110068**	**103**
**140R+141R**^b^	**121397**	**122311/124558**		93R	110141	113554	1137
143L	124882	124643	79	94L	113878	113639	79
144R	124963	125421	152	95R	113959	114417	152
145R	125480	125977	165	96R	114476	114973	165
146L	127052	126078	324	97L	116050	115076	324
147L	128221	127187	344	98L	117220	116186	344
148R	128324	128803	159	99R	117323	117802	159
149R	128843	129220	125	**99.5R**	**117842**	**118219**	**125**
150L	130827	129301	508	100L	119826	118300	508
151L	131435	130848	195	101L	120434	119847	195
152R	131534	132772	412	102R	120533	121771	412
153L	132661	132089	190	103L	121660	121088	190
154R	132788	133081	97	**103.5R**	**121787**	**122080**	**97**
155R	133172	134899	575	104R	122172	123896	574
156L	135860	135048	270	105L	124852	124043	269
157R	135948	136472	174	106R	124940	125464	174
158L	136944	136528	138	106.5L	125936	125520	138
159R	137020	137511	163	**106.7R**	**126012**	**126503**	**163**
160L	137996	137508	162	**107L**	**126988**	**126500**	**162**
161.5L	138598	138309	95	**107.5L**	**127561**	**127296**	**87**
162L	139822	138674	382	108L	128797	127649	382
**0.5L**	**1029**	**0/140141-140020**	**391**	109L	130138	128963	391
1L	1971	1057	304	110L	131080	130166	304
3R	2018	3163	381	111R	131127	132272	381
4L	4332	3235	365	113L	133442	132345	365
5L	5542	4400	380	114L	134652	133510	380
6R	5570	6349	259	115R	134680	135453	257
7L	7339	6416	307	116L	136425	135520	301
8L	7886	7194	230	117L	136972	136280	230
9L	8444	7980	154	118L	137530	137066	154
10L	8888	8517	123	**118.5L**	**137974**	**137603**	**123**
11L	9132	8944	62	119L	138218	138030	62
12L	12293	9219	1024	**120.5L**	**138307/139793**	**1/1540**	**1008**

^a^ORFs that have been added or altered are highlighted in bold. If a previously annotated ORF is not listed in the table, it has been deleted.

^b^Potentially frameshifted ORF

^c^Where an ORF has a potential sequencing error resulting in a frameshift mutation, 2 stop codons are provided in the format X/Y. The first number represents the actual physical stop in the reported sequence. The second number is the proposed stop if a sequencing error occurred.

^d^Length of ORF in amino acids

As seen above, our comparative genomic approach was able to identify previously unannotated ORFs, homologous ORFs with potential frameshifts, and ORFs split between the two ends of a circular genome. Although this approach proved extremely successful for the *Ranavirus *and *Megalocytivirus *genera, we were unable to use it for the *Chloriridovirus, Iridovirus*, and *Lymphocystivirus *genera. This is due to the lack of co-linearity and the highly divergent sets of genes that exist between the members of these genera, as well as the low number of available genome sequences. However, we did modify the annotations of lymphocystis disease virus-China (LCDV-China) and invertebrate iridescent virus-6 (IIV-6). The previous annotations of these genomes of both species had contained a large number of overlapping ORFs [2,24], which we decided to exclude on several grounds. First, LCDV-China and IIV-6 are the only iridoviruses, out of the twelve so far sequenced, in which overlapping ORFs have been annotated. In addition, the original sequencing paper for IIV-6 [2] and a follow-up paper by the same group [25] did not include a number of the overlapping ORFs reported in the database sequence, presumably due to their small size and lack of similarity with other viral and cellular genes. Finally, there is no experimental or bioinformatics evidence to suggest that any of these ORFs encode proteins. Therefore, to improve the overall consistency of the *Iridoviridae *family annotations, we removed the small overlapping ORF annotations from the LCDV-China and IIV-6 genomic sequences (Table 5, Additional File 1 &2).

**Table 5:** Overlapping ORFs deleted from the *Iridovirus *and *Lymphocystivirus *genera

**Virus**	**Deleted**
**LCDV-C**	4L, 8R, 17R, 20L, 21L, 26L, 28L, 30L, 31L, 32L, 35R, 36R, 44R, 46L, 48L, 52R, 55L, 68L, 74R, 76R, 78R, 79R, 81R, 88R, 92R, 94R, 98R, 102R, 103R, 113L, 120R, 130L, 132L, 134L, 138R, 141R, 144L, 152L, 155L, 156L, 163L, 167R, 174L, 183L, 188L, 192L, 193L, 194L, 195L, 198R, 199L, 200R, 204R, 207L, 210L, 213L, 223R, 225R, 232R, 233L, 236L, 238R, 240L
**IIV-6**	1R, 2R, 3R, 4R, 5R, 7R, 8R, 11L, 13R, 14R, 15R, 16L, 17R, 18R, 20L, 21R, 23L, 24L, 25R, 26R, 27L, 28L, 31R, 33L, 35L, 36R, 38R, 39R, 40R, 46R, 47R, 48R, 51R 52R, 53R, 54R, 55R, 57L, 58L, 59R, 63R, 64L, 66L, 68L, 70R, 72R, 73R, 74R, 76L, 78R, 79L, 80L, 81L, 86R, 87R, 88L, 89L, 90R, 91R, 92R, 93R, 97L, 99L, 102R, 103R, 105R, 108R, 109R, 112R, 114L, 119R, 124L, 125L, 128L, 129R, 131L, 133R, 134L, 144R, 147L, 150R, 151R, 152R, 153L, 154R, 158R, 163L, 164R, 166L, 167L, 168R, 171R, 173R, 174R, 177L, 178L, 180L, 181L, 182L, 183L, 185L, 186L, 187R, 188L, 189L, 190R, 191L, 194R, 199L, 202L, 204L, 207L, 108L, 210L, 214L, 215R, 217L, 220L, 222R, 223L, 230L, 231R, 233L, 237R, 239R, 243R, 245R, 248R, 252L, 256R, 257R, 258R, 260R, 262R, 263L, 264R, 265L, 266L, 267R, 269R, 270R, 271R, 275R, 276L, 277R, 278L, 279R, 280R, 281R, 282R, 283L, 286L, 288R, 290R, 291R, 292L, 294R, 296R, 297L, 298R, 299R, 303R, 304R, 305L, 310R, 311R, 314L, 316R, 318R, 319L, 320L, 321R, 323L, 324L, 326L, 327R, 328L, 330L, 331R, 333L, 334R, 336R, 338L, 339L, 341R, 344R, 345R, 351R, 352R, 353L, 354L, 355R, 356L, 360R, 362R, 363L, 364L, 365L, 367L, 370R, 371R, 372R, 377R, 379L, 381L, 382R, 383L, 386R, 387R, 390R, 392R, 394R, 397L, 398R, 399R, 402L, 403L, 405L, 406R, 407R, 408R, 409R, 410L, 412L, 416R, 417L, 418R, 419L, 421L, 424R, 425R, 427R, 429R, 430R, 431L, 432R, 433R, 434L, 435R, 440R, 442L, 444R, 445L, 446L, 447L, 448L, 449L, 450L, 452R, 455L, 456R, 459L, 461R, 462R, 464R, 465R

### Defining the conserved genes in Iridoviruses

**Figure 1:**
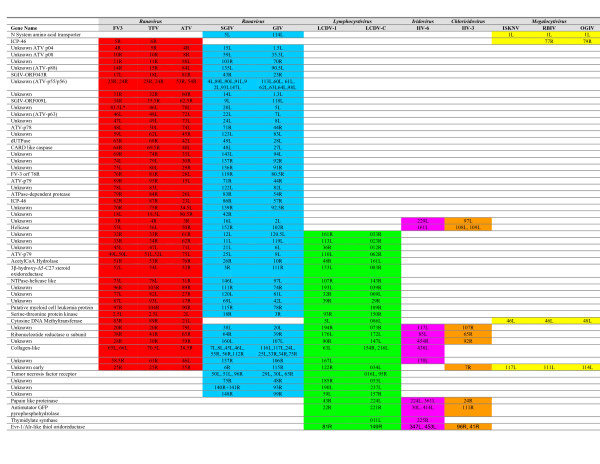
Conserved Iridovirus Genes. Every Iridoviridae gene that has an ortholog in at least 2 *Iridoviridae *genera are shown. Orthologs share the same row on the table. The genes within each genus are color-coded for easier identification. As long as at least one member of the genus contains an ortholog, the entire genus is highlighted. Where multiple ORFs are listed for a particular gene name, the ORFs represent multiple orthologs of the gene in that viral species. The remainder of the figure showing just the genes conserved between the Iridovirus and Chloriridovirus genera are included in Additional File 3.

As a result of this re-annotation of the *Iridoviridae *family, species within each genus now have a much greater consensus among their annotated ORFs. Prior to re-annotation, only 19 ORFs appeared to be conserved across all iridovirus species (Table 6). Although a previous report has suggested that 27 core genes exist within the *Iridoviridae *family [26], those core genes reported are found in most, but not all published iridoviridal species. In light of our previous results, we re-examined this core set of genes using the VOCs software. We identified seven novel core genes (Table 7), increasing the total number to 26 (Table 6 &7). This increase in the number of core genes was primarily due to the five new genes annotated during the re-analysis of RBIV (Table 7 bold highlighted genes). As expected most of the core genes are predicted to have essential functions, required for transcription, replication, and virus formation. Interestingly, three core genes, the orthologs of FV3 12L, 41R, and 94L, have no predicted functions. As previously stated Delhon et al. [26] identified 27 core genes, one more than we identified after our re-analysis. Delhon et al. [26] report the orthologs of FV3 20L represent a core [26]. However, our analysis shows that orthologs of FV3 20L exist in all genera except the Megalocytivirus (Figure 1) suggesting that FV3 20L is not a core gene. Future research to determine the functions of these genes, which are also likely to be essential, will provide important data for understanding the replication cycle of iridoviruses.

**Table 6:** *Iridoviridae *Core Genes

	**Gene Name**^a^	**FV3**	**TFV**	**ATV**	**SGIV**	**GIV**	**LCDV-1**	**LCDV-C**	**ISKNV**	**RBIV**	**OSGIV**	**IIV-6**	**MIV**
1.	Putative replication factor and/or DNA binding-packing	1R	105R	91R	116R	79R	162L	181R	61L	57L	60L	282R	79L
2.	DNA-dep RNA pol-II Largest subunit	8R	8R	6R	104L	71L	16L	191R	28L	29L	31L	176R, 343L	90L
3.	Putative NTPase I	9L	9L	7L	60R	36R	132L	075L	63L	59L	63L	22L	87L
4.	ATPase-like protein	15R	16R	83R	134L	90L	54R	114L	122R	116R	119R	75L	88R
5.	Helicase family	21L	21L	78R	54R	32R	6L	7L	56L	54L	57L	67R	4R
6.	D5 family NTPase involved in DNA replication	22R	22R	77L	52L	31L	128L	80L	109L	101L	106L	184R	121R
7.	Putative tyrosin kinase/lipopolysaccharide modifying enzyme	27R	29R	58R	**78L+81L**^b^	52L	195R	173R	61L, 114L	57L, **106L**^b^	60L, 111L	179R, 439L	35R
8.	NIF-NLI interacting factor	37R	40R	64R	61R	37R	82L	148L	5L	6L	6L	355R	104R
9.	Unknown	41R	45R	69R	57L	35L	163R	235R	76L	72L	75L	295L	16R
10.	Myristilated membrane protein	53R	55R	51L	88L	59L	67L	158R	7L	8L	8L	118L, 458R	6R
11.	DNA pol Family B exonuclease	60R	63R	44L	128R	87R	135R	203L	19R	20R	22R	37L	120L
12.	DNA-dep RNA pol-II second largest subunit	62L	65L	43R	73L	46L	25L	25R	34R	33R	36R	428L	9R
13.	Ribonucleotide reductase small subunit	67L	71L	38R	47L	26L	27R	41L	24R	26R	27R	376L	48L
14.	Ribonuclease III	80L	85L	25R	84L	55L	137R	187R	87R	83R	85R	142R	101R
15.	Proliferating cell nuclear antigen	84R	90R	20L	68L	41L	3L	197L	112R	**103R**^b^	109R	436L	60L
16.	Major capsid protein	90R	96R	14L	72R	45R	147L	43L	6L	7L	7L	274R	14L
17.	Putative XPPG-RAD2-type nuclease	95R	101R	10L	97L	66L	191R	169R	27L	28L	30L	369L	76L
18.	Serine-threonine protein kinase	19R	19R	80L	39L	21L	10L	45R	55L	53L	56L	380R	10L
19.	Serine-threonine protein kinase	57R	59R	47L	150L	100L	143L	178L	13R	13R	15R	98R	98L

The *Iridoviridae *core genes are shown.

^a^ORFs that have been added or altered are highlighted in bold

^b^Potentially frameshifted ORF

**Table 7:** Additional *Iridoviridae *Core Genes Identified After Genome Re-analysis

	**Newly Characterized Gene Name**^a^	**FV3**	**TFV**	**ATV**	**SGIV**	**GIV**	**LCDV-1**	**LCDV-C**	**ISKNV**	**RBIV**	**OSGIV**	**IIV-6**	**MIV**
1.	Myristilated membrane protein	2L	**2L**^b^	1L	19R	4R	160L	38R	**90.5L**	85L	**88.5L**	337L	47R
2.	Unknown	12L	12L	87R	118R	80R	108L	100L	96L	**89.5L**^b^	93L	287R	56L
3.	Transcription elongation factor TFIIS	81R	86R	24L	85R	56R	171R	115R	29L	**29.5L**^b^	32L	349L	55R
4.	Deoxynucleoside kinase	85R	**91.5R**	19L	67L	40L	136R	027R	32R	31R	34R	143R	29R
5.	Erv1/Alr family	88R	94R	16L	70R	43R	106L	142L	43L	**43.5L**	45L	347L	96R
6.	Immediate early protein ICP-46	91R	97R	13L	162L	108L	47L	162R	115R	**108.5R**	112R	393L	39R
7.	Hypothetical protein-Clostridium tetani	94L	100R	11L	98R	67R	19R	153L	86R	**82.5R**	**84.5L**	307L	33L

The *Iridoviridae *core genes are shown.

^a^ORFs that have been added or altered are highlighted in bold

^b^Potentially frameshifted ORF

**Figure 2:**
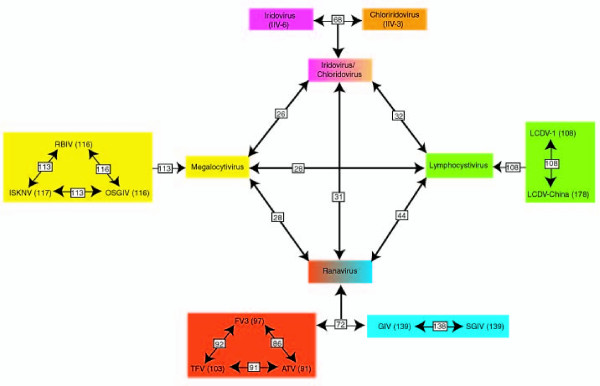
Phylogenetic relationships between the five iridovirus genera based on gene content. Individual viral species were compared within a genus to identify the number of orthologous genes. Orthologous genes between viral genera were then determined. The numbers on each line identify the number of orthologous genes shared between viral species or genera including the 26 core genes. The *Iridovirus *and *Chloriridovirus *genera have a high degree of gene conservation and a combined genera box (*Iridovirus*/*Chloriridovirus*) was used to compare orthologous genes between genera. In addition, two subgroups of the *Ranavirus *genus are shown. Each subgroup contains a virtually identical complement of genes. However, a comparison between the FV3/TFV/ATV subgroup with the SGIV/GIV subgroup revealed 72 orthologous genes.

Identifying genes conserved between some, but not all, iridovirus species can give us important information when investigating evolutionary relationships within the family. A number of past phylogenetic analyses of *Iridoviridae *have used phylogenic trees constructed from aligned protein sequences [1,18-20,22,24,27]. However, there are potential problems with phylogenic analysis based on comparisons of single genes. This type of analysis is rarely consistent due to horizontal gene transfer [28] and variable rates of evolution [29]. Therefore, we decided to take a whole genome comparative phylogenetic analysis to understand the relationship between iridoviruses. Our approach was to identify all the genes conserved between different genera to gain a better understanding of the relationships within the iridovirus family. This approach yields an indication of how similar in gene content 2 genomes are. Our whole-genome comparative analysis, grouped orthologous genes between genera (Figures 1 &2 and Additional File 3), and was consistent with phylogenic trees constructed from single protein sequences. Based on gene conservation, the *Ranavirus *and *Lymphocystivirus *genera appear to be most closely related to one another (Figure 2). In addition, the *Iridovirus *and *Chloriridovirus *genera are also closely related to one another based on presence of orthologous genes (Figure 2). In contrast, the *Megalocytivirus *genus and the *Iridovirus*/*Chloriridovirus *genera are equally divergent from each other as well as all other *Iridoviridae *family members (Figure 2).

**Figure 3:**
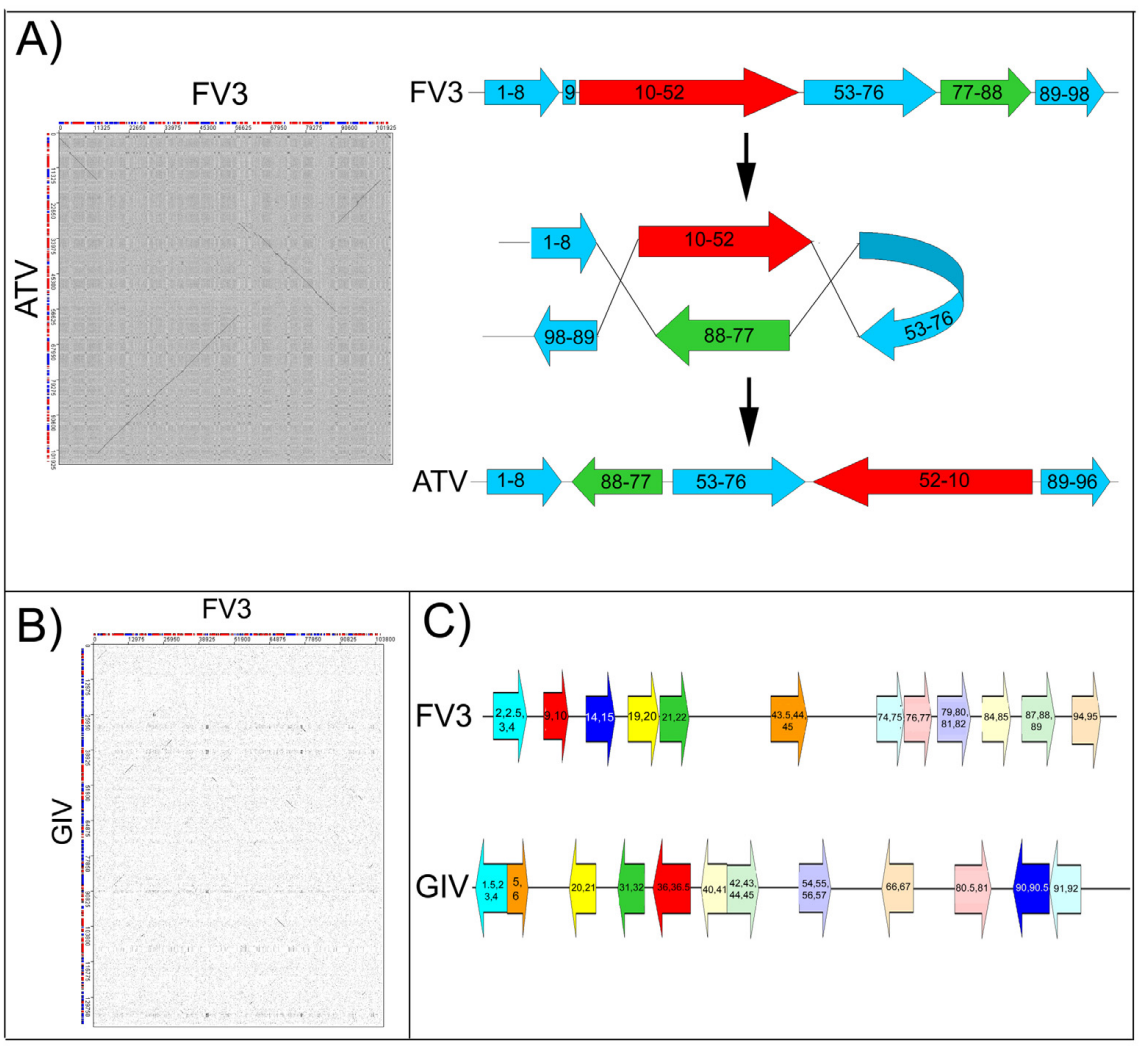
Co-linearity found within the *Ranavirus *genus. (A) FV3 and ATV, both members of the *Ranavirus *genus possess almost complete co-linearity of orthologous genes as visualized by a dotplot. However, 2 inversions have occurred. The FV3 genes 10–52 and 77–88 have switched genomic locations as shown, potentially through two recombination events. The inversion has also resulted in the loss of the ortholog of FV3 9L in ATV. (B) There is a limited amount of co-linearity found between FV3/TFV/ATV and SGIV/GIV. The co-linearity has been visualized using a dotplot analysis between FV3 (horizontal sequence) and GIV (vertical sequence). Genes are colored either red or blue representing right- or left-ward transcription respectively. (C) The co-linearity between FV3 and GIV is generally composed of stretches of 2 or 3 co-linear orthologous genes. Orthologous genes, in a co-linear arrangement are schematically shown as blocks of the same color on either FV3 or GIV genomic sequence.

As the list of sequenced iridovirus genomes grows, the non-co-linearity between many of these genomes becomes more apparent. The *Megalocytivirus and Ranavirus*, but not the *Chloriridovirus, Iridovirus*, and *Lymphocystivirus *genera, show a co-linear arrangement of genes within each genus. However, comparisons of genomic sequences from different genera suggest no co-linearity. This trend may be the result of the high recombination rates [30] seen in some iridovirus members [31]. For example, within the *Ranavirus *genus, ATV has two inversions relative to the FV3 and TFV sequences [30], reducing the co-linearity of these genomes to some degree. Figure 3A shows how two recombination events could convert FV3 to the ATV arrangement of genes. In contrast, a comparison between the more distantly related members within the *Ranavirus *genus (such as FV3 and GIV) demonstrate a much more dramatic loss of co-linearity. No long stretches of co-linear genes exist between these sequences, although small sections of co-linearity remain as seen through a dotplot analysis between FV3 and GIV (Figure 3B). The dotplot shows small regions of co-linearity scattered throughout the genome of FV3 and GIV as seen by short diagonal lines on the dotplot (Figure 3B). A schematic representation of the co-linearity between FV3 and GIV demonstrates that co-linearity occurs in small clusters of genes often only 2–4 genes in length (Figure 3C).

## Conclusion

The *Iridoviridae *family can cause severe diseases resulting in significant economic and environmental losses. Very little is known about how iridoviruses cause disease in their host. Our re-analysis of genomes within the *Iridoviridae *family provides a unifying framework to understand the biology of these viruses. For example, the re-analysis of the *Iridoviridae *family has increased the consistency of annotated sequences from viruses within the same genus. In addition, the re-analysis has helped create a much greater consensus among *Iridoviridae *family members and enhanced our understanding of this virus family as a whole. The updated annotations that we have produced for the iridovirus sequences can be found in the additional files to this paper; in addition, the databases and tools to analyse *Iridoviridae *genomes are available to all researchers [32]. This database will contain genomes from the original GenBank files and also the edited genomes described in this paper. Further re-defining the core set of iridovirus genes will continue to lead us to a better understanding of the phylogenetic relationships between individual iridoviruses as well as giving us a much deeper understanding of iridovirus replication. In addition, this analysis will provide a better framework for characterizing and annotating currently unclassified iridoviruses.

## Methods

### Re-annotation of the iridoviridae

Annotated sequences for the twelve completely sequenced iridovirus genomes (Table 1) were obtained from GenBank files and imported into the Viral Orthologous Clusters (VOCs) database [15]. Species from the same genus were examined using VOCs to identify all of the orthologous genes. The analysis then focused on the differences found between genomes within the same genus. For those genomes that contained co-linear arrangements of genes (those in the *Ranavirus *and *Megalocytivirus genera*), we compared those regions containing annotated ORFs. If more than two sequenced genomes were available for a given genus, and the ORF was present in at least two of the genomes, then we set out to determine if that ORF was also present in the remainder of the genomes. By this method, we were able to re-annotate small segments of each genome without needing to re-analyse the entire genome. The Viral Genome Organizer (VGO) software [16] was used to visualize the annotated ORFs, as well as the start and stop codons found within each genome.

### Analysis of orthologous genes

We used a combination of BLAST searches and queries using the VOCs software [32] to define orthologous genes between *Iridoviridae *genera. VOCs is a JAVA client-server that accesses a sequence query language (SQL) database containing iridovirus genomes. This SQL database permits complex queries to be assembled in an easy to use graphical user interface. VOCs initially groups orthologous genes into families based on BLASTP scores, these can be manually checked and altered if necessary.

### Dotplot analysis

Dotplots of FV3 and GIV were done using JDotter [33]. JDotter provides an interactive input window that links JDotter to the VOCs database. The sequences for the FV3 and GIV were obtained through the VOCs database.

## Competing interests

The author(s) declare that there are no competing interests.

## Authors' contributions

HEE, JM, EP, and CRB carried out the analysis of the *Iridoviridae *family and generated the tables and figures. VTJ and CU generated the databases and tools to carry out the analysis done in the manuscript. CRB and CU conceived of the study, and participated in its design and coordination and helped to draft the manuscript. All authors read and approved the final manuscript.

## Supplementary Material

Additional File 1 Revised annotation of the *Lymphocystivirus *genus. The table highlights the changes made to the *Lymphocystivirus *genus. Click here for file

Additional File 2 Analysis of the *Iridovirus *and *Chloriridovirus *genera. The table highlights the changes made to the *Iridovirus *and *Chloriridovirus *genera. Click here for file

Additional File 3 Additional Conserved Genes Between Iridovirus & Chloriridovirus genera. The table is an extension of Figure 1 – showing the genes that are conserved just between the Iridovirus and Chloriridovirus genera. Click here for file
