# Supplementary material for: Comparative genomic analysis of the family Iridoviridae: re-annotating and defining the core set of iridovirus genes
Source: Virol J. 2007 Jan 19;4:11. doi: 10.1186/1743-422X-4-11 (PMC1783846; doi:10.1186/1743-422X-4-11)
Supplement: Additional File 1 — Revised annotation of the Lymphocystivirus genus. The table highlights the changes made to the Lymphocystivirus genus. [file 1743-422X-4-11-S1.doc]

Revised annotation of the *Lymphocystivirus* genus

| **LCDV-1** | **Start** | **Stop** | **aa**^a^ | **LCDV-China** | **Start** | **Stop** | **aa**^a^ |
| --- | --- | --- | --- | --- | --- | --- | --- |
| 101L | 47032 | 46787 | 81 | 182R | 143285 | 143524 | 80 |
| 105L | 48233 | 47133 | 366 | 140L | 102507 | 101458 | 350 |
| 106L | 48786 | 48349 | 145 | 142L | 102988 | 102554 | 145 |
| 107R | 48830 | 49774 | 314 | 143R | 103037 | 103993 | 319 |
| 108L | 50719 | 49832 | 295 | 100L | 74643 | 73771 | 291 |
| 109L | 51830 | 50799 | 343 | 104L | 77978 | 76956 | 341 |
| 10L | 4271 | 2718 | 517 | 045R | 33396 | 34670 | 425 |
| 110L | 52586 | 51984 | 200 | 062R | 46203 | 46793 | 197 |
| 111L | 53669 | 52650 | 339 | 072R | 53025 | 54020 | 332 |
| 111L | 53669 | 52650 | 339 | 131R | 96217 | 97197 | 327 |
| 112L | 54107 | 53787 | 106 | 024R | 16464 | 116793 | 110 |
| 113L | 54309 | 54142 | 55 | 023R | 16260 | 16424 | 55 |
| 114L | 54736 | 54344 | 130 | 136L | 100198 | 99839 | 120 |
| 115L | 55256 | 54759 | 165 | 067R | 49321 | 50020 | 230 |
| 119L | 56008 | 55823 | 62 | 018L | 13143 | 12955 | 63 |
| 11R | 4355 | 5329 | 324 | 117L | 88281 | 87349 | 311 |
| 122R | 56274 | 56987 | 237 | 034L | 26151 | 25423 | 243 |
| 128L | 59907 | 57283 | 874 | 080L | 61359 | 58765 | 865 |
| 132L | 62818 | 59984 | 944 | 075L | 58165 | 55331 | 945 |
| 134L | 63283 | 62921 | 120 | 205R | 161257 | 161703 | 149 |
| 135R | 63353 | 66151 | 932 | 203L | 161193 | 158389 | 935 |
| 136R | 66272 | 66859 | 195 | 027R | 20577 | 21164 | 196 |
| 137R | 66939 | 67694 | 251 | 187R | 146942 | 147694 | 251 |
| 138R | 67741 | 68406 | 221 | 022R | 15055 | 15702 | 216 |
| 139R | 68512 | 68901 | 129 | 112R | 83202 | 83735 | 178 |
| 139R | 68512 | 68901 | 129 | 145R | 104543 | 104932 | 130 |
| 143L | 70394 | 68991 | 467 | 178L | 139562 | 138150 | 471 |
| 144L | 70779 | 70462 | 105 | 057R | 41721 | 42020 | 100 |
| 145R | 70802 | 71332 | 176 | 056L | 41699 | 41133 | 189 |
| 147L | 72774 | 71395 | 459 | 043L | 32783 | 31407 | 459 |
| 151L | 74781 | 74128 | 217 | 231L | 176937 | 176209 | 243 |
| 152R | 74926 | 75408 | 160 | 234R | 176981 | 177439 | 153 |
| 153L | 76477 | 75458 | 339 | 003R | 1911 | 2930 | 340 |
| 154R | 76704 | 77612 | 302 | 087L | 66826 | 65909 | 306 |
| 157L | 78493 | 77708 | 261 | 133R | 97712 | 98722 | 337 |
| 157L | 78493 | 77708 | 261 | 179L | 140985 | 140065 | 307 |
| 158L | 80009 | 78543 | 488 | 185R | 144571 | 146019 | 483 |
| 160L | 81193 | 80159 | 344 | 038R | 26690 | 27619 | 310 |
| 161R | 81357 | 82688 | 443 | 033R | 23615 | 24976 | 454 |
| 162L | 83446 | 82691 | 251 | 181R | 142378 | 143133 | 252 |
| 163R | 83522 | 86779 | 1085 | 235R | 177645 | 180923 | 1093 |
| 167L | 87577 | 86774 | 267 | 101L | 75803 | 74982 | 274 |
| 168R | 87651 | 87989 | 112 | 099L | 73633 | 73307 | 109 |
| 168R | 87651 | 87989 | 112 | 217R | 168693 | 169028 | 112 |
| 169R | 87994 | 88197 | 67 | 218R | 169041 | 169225 | 62 |
| 16L | 9078 | 5479 | 1199 | 191R | 150198 | 153776 | 1193 |
| 170L | 89572 | 88235 | 445 | 019R | 13401 | 14729 | 443 |
| 171R | 89590 | 89859 | 89 | 115R | 85683 | 85919 | 79 |
| 172L | 90068 | 89868 | 66 | 069L | 50841 | 50647 | 65 |
| 176L | 91733 | 90090 | 547 | 172L | 130914 | 129274 | 547 |
| 17R | 9192 | 10205 | 337 | 059R | 44029 | 45015 | 329 |
| 17R | 9192 | 10202 | 337 | 104L | 77978 | 76956 | 341 |
| 180L | 93899 | 92502 | 465 | 082R | 61859 | 62260 | 134 |
| 180L | 93899 | 92502 | 465 | 107L | 80761 | 79595 | 389 |
| 180L | 93899 | 92502 | 465 | 160R | 117323 | 118495 | 391 |
| 181R | 93998 | 94351 | 117 | 093L | 70333 | 69947 | 129 |
| 182R | 94402 | 94848 | 148 | 091L | 69880 | 69479 | 134 |
| 185R | 95482 | 96120 | 212 | 053L | 40118 | 39549 | 190 |
| 186L | 95532 | 95002 | 176 | 054R | 40140 | 40667 | 176 |
| 190L | 97947 | 96193 | 584 | 237L | 184512 | 181744 | 923 |
| 191R | 98034 | 99035 | 333 | 169R | 125950 | 126960 | 337 |
| 193L | 99876 | 99160 | 238 | 039R | 28016 | 28684 | 223 |
| 194R | 99948 | 100349 | 133 | 073R | 54512 | 54916 | 135 |
| 195R | 100468/102651 | 263/622 | 935 | 173R | 131370 | 134168 | 933 |
| 19R | 10234 | 10722 | 162 | 153L | 110936 | 110439 | 166 |
| 21R | 10740 | 11384 | 214 | 239R | 184871 | 186256 | 462 |
| 22R | 11776 | 12219 | 147 | 221R | 170159 | 170605 | 149 |
| 24R | 15254 | 15697 | 147 | 066R | 48716 | 49072 | 119 |
| 25L | 15290 | 12216 | 1024 | 025R | 17018 | 20068 | 1017 |
| 27R | 15868 | 17004 | 378 | 041L | 30804 | 29683 | 374 |
| 32R | 17027 | 17314 | 95 | 009L | 6022 | 5741 | 94 |
| 35L | 17616 | 17311 | 101 | 014L | 10927 | 10643 | 95 |
| 36R | 17725 | 18111 | 128 | 012R | 7733 | 8116 | 128 |
| 37L | 18576 | 18103 | 157 | 151R | 109535 | 110014 | 160 |
| 38L | 18832 | 18554 | 92 | 128L | 94858 | 94586 | 91 |
| 39R | 19001 | 20755 | 584 | 29R | 21472 | 23208 | 579 |
| 3L | 1365 | 619 | 248 | 197L | 154686 | 153940 | 249 |
| 42L | 21138 | 20782 | 118 | 226R | 172401 | 172751 | 117 |
| 43R | 21169 | 22461 | 430 | 224L | 172370 | 171090 | 427 |
| 47L | 23643 | 22519 | 374 | 162R | 120803 | 121969 | 389 |
| 48R | 23711 | 25579 | 622 | 161L | 120785 | 118915 | 624 |
| 50L | 26082 | 25576 | 168 | 065R | 47542 | 48042 | 167 |
| 52L | 26493 | 26266 | 75 | 040R | 29208 | 29441 | 78 |
| 54R | 27353 | 28087 | 244 | 114L | 84942 | 84211 | 244 |
| 58L | 28598 | 28092 | 168 | 006R | 3686 | 4183 | 166 |
| 59L | 29183 | 28722 | 153 | 157R | 114005 | 114463 | 153 |
| 5L | 2151 | 1468 | 228 | 086L | 64011 | 63688 | 108 |
| 63L | 30167 | 29232 | 311 | 154R | 112593 | 113570 | 326 |
| 63L | 30167 | 29232 | 311 | 216L | 168452 | 167850 | 201 |
| 64L | 30665 | 30300 | 121 | 159R | 116374 | 116736 | 121 |
| 67L | 32025 | 30646 | 459 | 158R | 115020 | 116390 | 457 |
| 69L | 32235 | 32768 | 178 | 112R | 83202 | 83735 | 178 |
| 6L | 2731 | 2237 | 164 | 007L | 5259 | 4765 | 165 |
| 70R | 32893 | 33507 | 204 | 111L | 83147 | 82530 | 206 |
| 72R | 33570 | 33971 | 133 | 122R | 91498 | 91881 | 128 |
| 73R | 34026 | 34460 | 144 | 070L | 51380 | 50925 | 152 |
| 79L | 36769 | 35477 | 430 | 119L | 90542 | 89130 | 471 |
| 80R | 36865 | 38421 | 519 | 013L | 10058 | 8501 | 519 |
| 81R | 39057 | 39491 | 144 | 149R | 106822 | 107196 | 125 |
| 82L | 39102 | 38572 | 176 | 148L | 106813 | 106268 | 182 |
| 86R | 40067 | 40330 | 87 | 212L | 166664 | 166446 | 73 |
| 87L | 40098 | 39502 | 198 | 214R | 166685 | 167239 | 185 |
| 88R | 40379 | 41893 | 504 | 150R | 107401 | 108923 | 506 |
| 90R | 41940 | 42389 | 149 | 147L | 105998 | 105555 | 148 |
| 91R | 42391 | 42795 | 134 | 146L | 105550 | 105146 | 135 |
| 93R | 42862 | 44055 | 397 | 201L | 156417 | 155221 | 399 |
| 93R | 42862 | 44055 | 397 | 175R | 134663 | 136102 | 480 |
| 94R | 44141 | 46732 | 863 | 166L | 125548 | 123146 | 801 |
|  |  |  |  | 001L | 649 | 524 | 42 |
|  |  |  |  | 002L | 1661 | 1362 | 100 |
|  |  |  |  | 005R | 2992 | 3243 | 84 |
|  |  |  |  | 010L | 6508 | 6371 | 46 |
|  |  |  |  | 011L | 7532 | 6675 | 286 |
|  |  |  |  | 015L | 11410 | 11264 | 49 |
|  |  |  |  | 016L | 12480 | 11647 | 278 |
|  |  |  |  | 037L | 26497 | 26372 | 42 |
|  |  |  |  | 042L | 31328 | 31197 | 44 |
|  |  |  |  | 047R | 35058 | 36179 | 374 |
|  |  |  |  | 049R | 36463 | 36891 | 143 |
|  |  |  |  | 050R | 37368 | 37517 | 50 |
|  |  |  |  | 051L | 38984 | 37536 | 483 |
|  |  |  |  | 058L | 43529 | 42531 | 333 |
|  |  |  |  | 060R | 45232 | 45366 | 45 |
|  |  |  |  | 061R | 45414 | 45923 | 170 |
|  |  |  |  | 063L | 47011 | 46892 | 40 |
|  |  |  |  | 064L | 47515 | 47348 | 56 |
|  |  |  |  | 071L | 52213 | 51614 | 200 |
|  |  |  |  | 077R | 58523 | 58762 | 80 |
|  |  |  |  | 083R | 62853 | 63008 | 52 |
|  |  |  |  | 084R | 63034 | 63153 | 40 |
|  |  |  |  | 085L | 63384 | 63229 | 52 |
|  |  |  |  | 089R | 67518 | 67647 | 43 |
|  |  |  |  | 090L | 68950 | 68408 | 181 |
|  |  |  |  | 095R | 70395 | 71261 | 289 |
|  |  |  |  | 096R | 71776 | 71934 | 53 |
|  |  |  |  | 097L | 72846 | 72037 | 270 |
|  |  |  |  | 105R | 78572 | 78709 | 46 |
|  |  |  |  | 106L | 79313 | 78897 | 139 |
|  |  |  |  | 108L | 80916 | 80767 | 50 |
|  |  |  |  | 109L | 81476 | 81324 | 51 |
|  |  |  |  | 110R | 81572 | 81934 | 121 |
|  |  |  |  | 116R | 86212 | 87081 | 290 |
|  |  |  |  | 118R | 88741 | 88860 | 40 |
|  |  |  |  | 121R | 90758 | 91093 | 112 |
|  |  |  |  | 123L | 92090 | 91935 | 52 |
|  |  |  |  | 124R | 92394 | 93368 | 325 |
|  |  |  |  | 125R | 93740 | 93874 | 45 |
|  |  |  |  | 126R | 93954 | 94124 | 57 |
|  |  |  |  | 127L | 94383 | 94246 | 46 |
|  |  |  |  | 129R | 94922 | 95392 | 157 |
|  |  |  |  | 135R | 99061 | 99660 | 200 |
|  |  |  |  | 137L | 101109 | 100627 | 161 |
|  |  |  |  | 139L | 101415 | 101275 | 47 |
|  |  |  |  | 164L | 122674 | 122075 | 200 |
|  |  |  |  | 165L | 123025 | 122816 | 70 |
|  |  |  |  | 168L | 125867 | 125739 | 43 |
|  |  |  |  | 170R | 127874 | 128008 | 45 |
|  |  |  |  | 171L | 128623 | 128483 | 47 |
|  |  |  |  | 176R | 136165 | 136347 | 61 |
|  |  |  |  | 177R | 137000 | 137404 | 135 |
|  |  |  |  | 180R | 141161 | 142072 | 304 |
|  |  |  |  | 184L | 144548 | 144423 | 42 |
|  |  |  |  | 186R | 146201 | 146539 | 113 |
|  |  |  |  | 189R | 148578 | 149099 | 174 |
|  |  |  |  | 190L | 149785 | 149483 | 101 |
|  |  |  |  | 196L | 153897 | 153775 | 41 |
|  |  |  |  | 202L | 157950 | 157021 | 310 |
|  |  |  |  | 208L | 163448 | 163005 | 148 |
|  |  |  |  | 209R | 163864 | 165933 | 690 |
|  |  |  |  | 211R | 166014 | 166280 | 89 |
|  |  |  |  | 215L | 167845 | 167714 | 44 |
|  |  |  |  | 219R | 169337 | 169600 | 88 |
|  |  |  |  | 220R | 169862 | 169990 | 43 |
|  |  |  |  | 222L | 171030 | 170830 | 67 |
|  |  |  |  | 227L | 172966 | 172838 | 43 |
|  |  |  |  | 228R | 173468 | 173887 | 140 |
|  |  |  |  | 229R | 174258 | 174627 | 124 |
|  |  |  |  | 230L | 174853 | 174713 | 47 |

^a^Length of ORF in amino acids
