# Supplementary material for: Comparative genomic analysis of the family Iridoviridae: re-annotating and defining the core set of iridovirus genes
Source: Virol J. 2007 Jan 19;4:11. doi: 10.1186/1743-422X-4-11 (PMC1783846; doi:10.1186/1743-422X-4-11)
Supplement: Additional File 2 — Analysis of the Iridovirus and Chloriridovirus genera. The table highlights the changes made to the Iridovirus and Chloriridovirus genera. [file 1743-422X-4-11-S2.doc]

Analysis of the *Iridovirus* and *Chloriridovirus* genera

| Iridovirus^a^ | | | | Chloriridovirus^a^ | | | |
| --- | --- | --- | --- | --- | --- | --- | --- |
| **IIV-6** | **Start** | Stop | **aa^b^** | **IIV-3** | **Start** | Stop | **aa^b^** |
| 378R | 167305 | 167883 | 193 | 100L | 156721 | 157359 | 212 |
| 142R | 55500 | 56375 | 292 | 101R | 157391 | 158392 | 333 |
| 355R | 158160 | 158705 | 182 | 104L | 161688 | 162248 | 186 |
| 30L | 11520 | 9931 | 530 | 106R | 163297 | 164718 | 473 |
| 117L | 46517 | 45771 | 249 | 107R | 164859 | 165647 | 262 |
| 161L | 66562 | 65237 | 442 | 108L+109L^c^ | 167108 | 165751 |  |
| 380R | 167936 | 169519 | 528 | 10L | 15859 | 17538 | 559 |
| 414L | 184146 | 183571 | 192 | 111R | 168970 | 169479 | 169 |
| 155L | 62770 | 62006 | 255 | 113L | 169940 | 172363 | 807 |
| 342R | 153531 | 153794 | 88 | 115R | 173073 | 173303 | 76 |
| 37L | 16288 | 12470 | 1273 | 120R | 179618 | 183043 | 1141 |
| 184R | 79271 | 82183 | 971 | 121R | 183279 | 186104 | 941 |
| 302L | 141139 | 140006 | 378 | 12R | 20020 | 21141 | 373 |
| 274R | 128758 | 130158 | 467 | 14L | 21706 | 23106 | 466 |
| 295L | 139425 | 135397 | 1343 | 16R | 26289 | 29708 | 1139 |
| 335L | 151418 | 150426 | 331 | 17R | 29876 | 30745 | 289 |
| 415R | 184256 | 184981 | 242 | 18L | 31118 | 31615 | 165 |
| 196R | 83714 | 84235 | 174 | 20R | 33982 | 34479 | 165 |
| 361L | 162659 | 161034 | 542 | 24R | 38120 | 39595 | 491 |
| 350L | 157714 | 157094 | 207 | 26R | 40206 | 40886 | 226 |
| 143R | 56488 | 57072 | 195 | 29R | 45281 | 45862 | 193 |
| 307L | 142846 | 142256 | 197 | 33L | 47878 | 48462 | 194 |
| 77L | 33545 | 32607 | 313 | 34R | 48593 | 49191 | 266 |
| 179R | 75567 | 79124 | 1186 | 35R | 49517 | 52813 | 1098 |
| 98R | 39478 | 41283 | 602 | 38R | 57775 | 59415 | 546 |
| 393L | 175775 | 174414 | 454 | 39R | 59602 | 60933 | 443 |
| 453L | 205047 | 204634 | 138 | 41R | 61328 | 61717 | 129 |
| 136R | 53353 | 53886 | 178 | 42R | 61812 | 622912 | 159 |
| 10R | 2498 | 2857 | 120 | 43R | 62326 | 62520 | 64 |
| 229L | 107921 | 106593 | 443 | 46R | 64376 | 66175 | 599 |
| 337L | 152755 | 151520 | 412 | 47R | 66434 | 67657 | 407 |
| 376L | 167187 | 166108 | 360 | 48L | 67723 | 68853 | 376 |
| 67R | 29008 | 30375 | 456 | 4R | 5671 | 7026 | 451 |
| 145L | 57566 | 57102 | 155 | 50L | 71122 | 71580 | 152 |
| 213R | 98000 | 99565 | 522 | 51L | 74573 | 75421 | 282 |
| 205R | 89498 | 91342 | 615 | 52L | 75531 | 77801 | 756 |
| 349L | 157001 | 156582 | 140 | 55R | 83754 | 84167 | 137 |
| 287R | 132500 | 133444 | 315 | 56L | 84217 | 85251 | 344 |
| 391R | 173396 | 174277 | 294 | 58R | 85937 | 86389 | 150 |
| 12L | 4772 | 2901 | 624 | 59L | 86759 | 88441 | 560 |
| 436L | 193268 | 192354 | 305 | 60L | 88824 | 89678 | 284 |
| 467R | 210660 | 212168 | 503 | 61R | 94254 | 95720 | 488 |
| 85L | 37985 | 35109 | 959 | 65R | 97890 | 99782 | 630 |
| 197R | 84586 | 85383 | 266 | 67L | 101658 | 102380 | 240 |
| 401R | 179880 | 180608 | 243 | 68R | 102821 | 103426 | 201 |
| 198R | 85637 | 87052 | 472 | 69L | 103499 | 104770 | 423 |
| 118L | 48177 | 46633 | 515 | 6R | 8063 | 9547 | 494 |
| 306R | 141238 | 142176 | 312 | 70L | 104901 | 105749 | 282 |
| 259R | 120500 | 121396 | 299 | 71L | 105932 | 106597 | 221 |
| 268L | 127775 | 125646 | 710 | 74L | 107847 | 110351 | 834 |
| 369L | 164793 | 163549 | 415 | 76L | 113075 | 114217 | 380 |
| 244L | 115973 | 115128 | 282 | 78R | 116034 | 117077 | 347 |
| 282R | 130331 | 131479 | 383 | 79L | 117119 | 118306 | 395 |
| 232R | 108021 | 110033 | 671 | 84L | 121705 | 124239 | 844 |
| 45L | 21843 | 18448 | 1132 | 86L | 125214 | 128555 | 1113 |
| 22L | 9277 | 5765 | 1171 | 87L | 131107 | 134049 | 980 |
| 75L | 32451 | 31678 | 258 | 88R | 134089 | 134874 | 261 |
| 176R | 72297 | 75374 | 1026 | 90L | 135312 | 139445 | 1377 |
| 50L | 25767 | 22612 | 1052 | 94L | 144580 | 147069 | 829 |
| 165R | 67220 | 68011 | 264 | 95L | 147174 | 148265 | 363 |
| 347L | 156061 | 155729 | 111 | 96R | 148391 | 148837 | 148 |
| 170L | 69239 | 68502 | 246 | 97L | 150036 | 150653 | 205 |
| 439L | 196182 | 194572 | 537 | 98L | 150802 | 152364 | 520 |
| 329R | 148473 | 149606 | 378 | 99R | 155290 | 156678 | 462 |
| 428L | 192254 | 188676 | 1193 | 9R | 12359 | 15781 | 1140 |
| 100L | 41928 | 41383 | 181 |  |  |  |  |
| 101L | 42639 | 41938 | 234 |  |  |  |  |
| 104L | 43008 | 42838 | 57 |  |  |  |  |
| 106L | 43251 | 43081 | 57 |  |  |  |  |
| 107L | 43551 | 43312 | 80 |  |  |  |  |
| 110R | 43639 | 43845 | 68 |  |  |  |  |
| 111R | 43911 | 44435 | 175 |  |  |  |  |
| 113L | 44647 | 44465 | 61 |  |  |  |  |
| 115R | 44949 | 45314 | 122 |  |  |  |  |
| 116L | 45693 | 45409 | 95 |  |  |  |  |
| 120L | 48661 | 48302 | 120 |  |  |  |  |
| 121R | 48729 | 48995 | 89 |  |  |  |  |
| 122R | 49041 | 49463 | 141 |  |  |  |  |
| 123R | 49533 | 49958 | 142 |  |  |  |  |
| 126R | 50040 | 50861 | 273 |  |  |  |  |
| 127L | 51667 | 50963 | 235 |  |  |  |  |
| 130R | 51751 | 52353 | 201 |  |  |  |  |
| 132L | 53205 | 52489 | 239 |  |  |  |  |
| 137R | 53930 | 54100 | 57 |  |  |  |  |
| 138R | 54168 | 54509 | 114 |  |  |  |  |
| 139L | 54772 | 54578 | 65 |  |  |  |  |
| 140L | 55058 | 54867 | 64 |  |  |  |  |
| 141R | 55099 | 55350 | 84 |  |  |  |  |
| 146R | 57731 | 58465 | 245 |  |  |  |  |
| 148R | 58646 | 59887 | 414 |  |  |  |  |
| 149L | 61960 | 59963 | 666 |  |  |  |  |
| 156R | 62812 | 62982 | 57 |  |  |  |  |
| 157L | 63453 | 62998 | 152 |  |  |  |  |
| 159L | 64967 | 63543 | 475 |  |  |  |  |
| 160L | 65228 | 65070 | 53 |  |  |  |  |
| 162R | 66645 | 67100 | 152 |  |  |  |  |
| 169L | 68445 | 68050 | 132 |  |  |  |  |
| 172L | 71169 | 69352 | 606 |  |  |  |  |
| 175R | 71480 | 72031 | 184 |  |  |  |  |
| 192R | 82239 | 82457 | 73 |  |  |  |  |
| 193R | 82521 | 83144 | 208 |  |  |  |  |
| 195L | 83656 | 83240 | 139 |  |  |  |  |
| 19R | 4896 | 5732 | 279 |  |  |  |  |
| 200R | 87186 | 87368 | 61 |  |  |  |  |
| 201R | 87534 | 88790 | 419 |  |  |  |  |
| 203L | 89334 | 88855 | 160 |  |  |  |  |
| 206R | 91481 | 91936 | 152 |  |  |  |  |
| 209R | 92277 | 95417 | 1046 |  |  |  |  |
| 211L | 96600 | 95431 | 390 |  |  |  |  |
| 212L | 97815 | 96736 | 360 |  |  |  |  |
| 216R | 99596 | 100075 | 160 |  |  |  |  |
| 218R | 100283 | 101107 | 274 |  |  |  |  |
| 219L | 102489 | 101461 | 343 |  |  |  |  |
| 224L | 104051 | 102705 | 449 |  |  |  |  |
| 225R | 104095 | 104979 | 295 |  |  |  |  |
| 226R | 105047 | 105334 | 96 |  |  |  |  |
| 227L | 105597 | 105409 | 63 |  |  |  |  |
| 228L | 106532 | 105726 | 269 |  |  |  |  |
| 234R | 110163 | 110741 | 193 |  |  |  |  |
| 235L | 111580 | 110786 | 265 |  |  |  |  |
| 236L | 112077 | 111859 | 73 |  |  |  |  |
| 238R | 112084 | 113403 | 440 |  |  |  |  |
| 240R | 113666 | 113851 | 62 |  |  |  |  |
| 241L | 114570 | 114061 | 170 |  |  |  |  |
| 242L | 114902 | 114606 | 99 |  |  |  |  |
| 246L | 116397 | 116020 | 126 |  |  |  |  |
| 247L | 116827 | 116477 | 117 |  |  |  |  |
| 249R | 117165 | 117326 | 54 |  |  |  |  |
| 250L | 117787 | 117365 | 141 |  |  |  |  |
| 251L | 118553 | 117915 | 213 |  |  |  |  |
| 253L | 119140 | 118643 | 166 |  |  |  |  |
| 254L | 120368 | 119361 | 336 |  |  |  |  |
| 261R | 121519 | 125601 | 1361 |  |  |  |  |
| 272L | 128014 | 127802 | 71 |  |  |  |  |
| 273R | 128273 | 128680 | 136 |  |  |  |  |
| 284R | 131527 | 131970 | 148 |  |  |  |  |
| 285L | 132415 | 132053 | 121 |  |  |  |  |
| 289L | 134746 | 133496 | 417 |  |  |  |  |
| 293R | 134869 | 135324 | 152 |  |  |  |  |
| 29R | 9504 | 9890 | 129 |  |  |  |  |
| 300R | 139519 | 139701 | 61 |  |  |  |  |
| 301L | 139955 | 139734 | 74 |  |  |  |  |
| 308L | 143095 | 142877 | 73 |  |  |  |  |
| 309L | 143800 | 143150 | 217 |  |  |  |  |
| 312R | 143926 | 144201 | 92 |  |  |  |  |
| 313L | 145342 | 144269 | 358 |  |  |  |  |
| 315L | 146128 | 145433 | 232 |  |  |  |  |
| 317L | 147527 | 146274 | 418 |  |  |  |  |
| 322R | 147601 | 147807 | 69 |  |  |  |  |
| 325L | 148350 | 147871 | 159 |  |  |  |  |
| 32R | 11651 | 11953 | 100 |  |  |  |  |
| 332L | 150355 | 149654 | 234 |  |  |  |  |
| 340R | 152801 | 153319 | 173 |  |  |  |  |
| 343L | 155241 | 153856 | 462 |  |  |  |  |
| 346R | 153331 | 15537 | 69 |  |  |  |  |
| 348R | 156103 | 156396 | 98 |  |  |  |  |
| 34R | 11997 | 12401 | 134 |  |  |  |  |
| 352.5L | 157963 | 157881 | 27 |  |  |  |  |
| 357R | 158775 | 159266 | 164 |  |  |  |  |
| 358L | 159788 | 159333 | 152 |  |  |  |  |
| 359L | 160938 | 159901 | 346 |  |  |  |  |
| 366R | 162769 | 163110 | 114 |  |  |  |  |
| 368R | 163160 | 163441 | 93 |  |  |  |  |
| 373L | 165531 | 164803 | 243 |  |  |  |  |
| 374L | 166062 | 165568 | 165 |  |  |  |  |
| 384L | 169797 | 169675 | 40 |  |  |  |  |
| 385L | 170627 | 169866 | 254 |  |  |  |  |
| 388R | 170803 | 171843 | 347 |  |  |  |  |
| 389L | 173380 | 172148 | 411 |  |  |  |  |
| 395R | 175856 | 176308 | 151 |  |  |  |  |
| 396L | 179694 | 176524 | 1057 |  |  |  |  |
| 400R | 179736 | 179867 | 43 |  |  |  |  |
| 404L | 181442 | 180729 | 238 |  |  |  |  |
| 411L | 182736 | 181822 | 304 |  |  |  |  |
| 413R | 182811 | 183434 | 208 |  |  |  |  |
| 41L | 16745 | 16380 | 122 |  |  |  |  |
| 420R | 185306 | 186646 | 447 |  |  |  |  |
| 422L | 187279 | 186683 | 596 |  |  |  |  |
| 423L | 187883 | 187341 | 181 |  |  |  |  |
| 426R | 187960 | 188373 | 138 |  |  |  |  |
| 42R | 16811 | 17092 | 94 |  |  |  |  |
| 437L | 193538 | 193344 | 65 |  |  |  |  |
| 438L | 194534 | 193647 | 296 |  |  |  |  |
| 43L | 17690 | 17343 | 116 |  |  |  |  |
| 441R | 196413 | 196760 | 116 |  |  |  |  |
| 443R | 196872 | 204167 | 2432 |  |  |  |  |
| 44R | 18066 | 18260 | 65 |  |  |  |  |
| 451L | 204586 | 204197 | 129 |  |  |  |  |
| 454R | 205154 | 205705 | 184 |  |  |  |  |
| 457L | 206658 | 205777 | 294 |  |  |  |  |
| 458R | 207044 | 208528 | 495 |  |  |  |  |
| 460R | 208588 | 209247 | 220 |  |  |  |  |
| 463L | 210231 | 209359 | 290 |  |  |  |  |
| 466R | 210309 | 210578 | 90 |  |  |  |  |
| 468L | 212244 | 889 | 376 |  |  |  |  |
| 49L | 22304 | 21936 | 123 |  |  |  |  |
| 56R | 25855 | 26703 | 283 |  |  |  |  |
| 60L | 27449 | 26742 | 236 |  |  |  |  |
| 61R | 27538 | 28002 | 155 |  |  |  |  |
| 62L | 28607 | 28071 | 179 |  |  |  |  |
| 65R | 28749 | 28913 | 55 |  |  |  |  |
| 69L | 31144 | 30455 | 230 |  |  |  |  |
| 6L | 2105 | 1050 | 352 |  |  |  |  |
| 71L | 31614 | 31225 | 129 |  |  |  |  |
| 82L | 34404 | 33937 | 156 |  |  |  |  |
| 83L | 34553 | 34404 | 50 |  |  |  |  |
| 84L | 35091 | 34597 | 165 |  |  |  |  |
| 94L | 38433 | 38077 | 118 |  |  |  |  |
| 95L | 38784 | 38530 | 85 |  |  |  |  |
| 96L | 39395 | 38874 | 174 |  |  |  |  |
| 9R | 2207 | 2464 | 84 |  |  |  |  |
|  |  |  |  | 1R | 2620 | 3066 | 148 |
|  |  |  |  | 2R | 3603 | 4979 | 458 |
|  |  |  |  | 3L | 5168 | 5638 | 156 |
|  |  |  |  | 5L | 7214 | 7867 | 217 |
|  |  |  |  | 7R | 9683 | 11026 | 447 |
|  |  |  |  | 13L | 21298 | 21570 | 90 |
|  |  |  |  | 15R | 23196 | 23624 | 142 |
|  |  |  |  | 21L | 34527 | 35147 | 206 |
|  |  |  |  | 22L | 35191 | 35868 | 225 |
|  |  |  |  | 23R | 37466 | 37786 | 106 |
|  |  |  |  | 25R | 39626 | 40162 | 178 |
|  |  |  |  | 28R | 44228 | 45241 | 337 |
|  |  |  |  | 30L | 45951 | 46385 | 144 |
|  |  |  |  | 31R | 46406 | 46834 | 142 |
|  |  |  |  | 32R | 46927 | 47766 | 279 |
|  |  |  |  | 37L | 56935 | 57696 | 253 |
|  |  |  |  | 40R | 61046 | 61297 | 83 |
|  |  |  |  | 44L | 62708 | 63715 | 335 |
|  |  |  |  | 45R | 63932 | 64204 | 90 |
|  |  |  |  | 49R | 69009 | 71060 | 683 |
|  |  |  |  | 53L | 78217 | 78645 | 142 |
|  |  |  |  | 54L | 82655 | 83584 | 309 |
|  |  |  |  | 57L | 85357 | 85749 | 130 |
|  |  |  |  | 62L | 95543 | 96241 | 232 |
|  |  |  |  | 63R | 96289 | 96927 | 212 |
|  |  |  |  | 64L | 96985 | 97827 | 280 |
|  |  |  |  | 66L | 99857 | 100438 | 193 |
|  |  |  |  | 72L | 106672 | 107142 | 156 |
|  |  |  |  | 73R | 107238 | 107771 | 177 |
|  |  |  |  | 75R | 112290 | 112766 | 158 |
|  |  |  |  | 77R | 115523 | 115966 | 147 |
|  |  |  |  | 80R | 118504 | 119229 | 241 |
|  |  |  |  | 81L | 119783 | 120310 | 175 |
|  |  |  |  | 82L | 120312 | 120800 | 162 |
|  |  |  |  | 83L | 121075 | 121653 | 192 |
|  |  |  |  | 85L | 124581 | 125066 | 161 |
|  |  |  |  | 89L | 134925 | 135221 | 98 |
|  |  |  |  | 92R | 142975 | 143490 | 171 |
|  |  |  |  | 102R | 161028 | 161393 | 121 |
|  |  |  |  | 103L | 161449 | 161631 | 60 |
|  |  |  |  | 105R | 162408 | 163148 | 246 |
|  |  |  |  | 110R | 167222 | 167995 | 257 |
|  |  |  |  | 112R | 169526 | 169864 | 112 |
|  |  |  |  | 114L | 172479 | 172916 | 145 |
|  |  |  |  | 116R | 173407 | 174171 | 254 |
|  |  |  |  | 117L | 174305 | 174511 | 68 |
|  |  |  |  | 118R | 174533 | 174757 | 74 |
|  |  |  |  | 119R | 174807 | 175187 | 126 |
|  |  |  |  | 122R | 186194 | 186397 | 67 |
|  |  |  |  | 123L | 186446 | 186865 | 139 |
|  |  |  |  | 124R | 186910 | 187620 | 236 |
|  |  |  |  | 125R | 187711 | 188604 | 297 |
|  |  |  |  | 126R | 188648 | 188965 | 105 |

^a^Orthologous ORFs share the same row of the table

^b^Length of ORF in amino acids

^c^Potentially frameshifted ORF
