# Supplementary material for: Comparative genomic analysis of the family Iridoviridae: re-annotating and defining the core set of iridovirus genes
Source: Virol J. 2007 Jan 19;4:11. doi: 10.1186/1743-422X-4-11 (PMC1783846; doi:10.1186/1743-422X-4-11)
Supplement: Additional File 3 — Additional Conserved Genes Between Iridovirus & Chloriridovirus genera. The table is an extension of Figure 1 – showing the genes that are conserved just between the Iridovirus and Chloriridovirus genera. [file 1743-422X-4-11-S3.doc]

Additional Conserved Genes Between Iridovirus & Chloriridovirus genera

|  |  | ***Iridovirus*** | ***Chloriridovirus*** |
| --- | --- | --- | --- |
|  | **Gene Name** | **IIV-6** | **IIV-3** |
| 1. | Unknown | 10R | 43R |
| 2. | Exonuclease II | 12L | 59L |
| 3. | Zn binding signature | 136R | 42R |
| 4. | Unknown | 145L | 50L |
| 5. | Unknown | 155L | 113L |
| 6. | Helicase | 161L | 108L+109L* |
| 7. | Metalloprotease | 165R | 95L |
| 8. | RuvC-like resolvase | 170L | 97L |
| 9. | Thioredoxin | 196R | 20R |
| 10. | S/Y protein phosphatase | 197R | 67L |
| 11. | Unknown | 198R | 69L |
| 12. | NAD-dependent DNA ligase | 205R | 52L |
| 13. | Unknown | 213R | 51L |
| 14. | Unknown | 229L | 46R |
| 15. | OUT domain | 232R | 84L |
| 16. | Phosphoesterase | 244L | 78R |
| 17. | Unknown | 259R | 71L |
| 18. | Unknown | 268L | 74L |
| 19. | C2H2 zinc finger protein | 302L | 12R |
| 20. | SWIB/MDM2 domain | 306R | 70L |
| 21. | Helicase | 30L | 106R |
| 22. | Unknown | 329R | 99R |
| 23. | Unknown | 342R | 115R |
| 24. | VV.A1L-like transcription factor | 350L | 26R |
| 25. | Unknown | 378R | 100L |
| 26. | Unknown | 391R | 58R |
| 27. | HMG-like | 401R | 68R |
| 28. | Unknown | 415R | 18L |
| 29. | Protein kinase-like protein | 439L | 98L |
| 30. | DNA topoisomerase II | 45L | 86L |
| 31. | Unknown | 467R | 61R |
| 32. | P-loop nucleoside triphosphate | 50L | 94L |
| 33. | Zn finger protein | 77L | 34R |
| 34. | Unknown | 98R | 38R |

*Potentially frameshifted ORF
